# Improved BDF Relaying Scheme Using Time Diversity over Atmospheric Turbulence and Misalignment Fading Channels

**DOI:** 10.1155/2014/213834

**Published:** 2014-01-22

**Authors:** Antonio García-Zambrana, Carmen Castillo-Vázquez, Beatriz Castillo-Vázquez

**Affiliations:** ^1^Department of Communications Engineering, University of Málaga, 29071 Málaga, Spain; ^2^Department of Statistics and Operations Research, University of Málaga, 29071 Málaga, Spain

## Abstract

A novel bit-detect-and-forward (BDF) relaying scheme based on repetition coding with the relay is proposed, significantly improving the robustness to impairments proper to free-space optical (FSO) communications such as unsuitable alignment between transmitter and receiver as well as fluctuations in the irradiance of the transmitted optical beam due to the atmospheric turbulence. Closed-form asymptotic bit-error-rate (BER) expressions are derived for a 3-way FSO communication setup. Fully exploiting the potential time-diversity available in the relay turbulent channel, a relevant better performance is achieved, showing a greater robustness to the relay location since a high diversity gain is provided regardless of the source-destination link distance.

## 1. Introduction

Atmospheric free-space optical (FSO) transmission using intensity modulation and direct detection (IM/DD) can be considered as an important alternative to consider for next generation broadband in order to support large bandwidth, excellent security, and quick setup [1]. However, this technology is not without drawbacks. Atmospheric turbulence causes fluctuations in the irradiance of the transmitted optical beam, which is known as *atmospheric scintillation*, severely degrading the link performance [2]. Since FSO systems are usually installed on high buildings, building sway causes vibrations in the transmitted beam, leading to an unsuitable alignment between transmitter and receiver and, hence, a greater deterioration in performance. Cooperative transmission can significantly improve the performance by creating diversity using the transceivers available at the other nodes of the network. Recently, several works have investigated the adoption of this technique in the context of FSO systems [3–9], being recognized as a very promising solution for future ad-hoc optical wireless systems. In [4, 5] a 3-way FSO communication setup is proposed to implement a cooperative protocol in order to improve spatial diversity without much increase in hardware. In [9], following the bit-detect-and-forward (BDF) cooperative protocol presented in [4], the analysis is extended to FSO communication systems using IM/DD over atmospheric turbulence and misalignment fading channels, considering cooperative communications with BDF relaying and equal gain combining (EGC) reception. In this paper, a novel BDF relaying scheme using time-diversity in the relay channel is proposed. Closed-form asymptotic bit error-rate (BER) expressions are obtained for a 3-way FSO communication setup, showing a significant improvement in the robustness to impairments proper to these systems such as unsuitable alignment between transmitter and receiver as well as fluctuations in the irradiance of the transmitted optical beam due to the atmospheric turbulence. Fully exploiting the potential time-diversity available in the relay turbulent channel, the superiority of the proposed scheme is corroborated if compared with the traditional BDF cooperative protocol [4, 9], since a greater robustness is provided to the relay location, maintaining a high diversity gain regardless of the source-destination link distance. In contrast to the traditional BDF, wherein the cooperative strategy works in two phases or transmission frames and the relay node detects each code bit of the cooperative signal individually and forwards it to the destination, the novel BDF relaying scheme here proposed is based on a new cooperative protocol working in three phases that implements time-diversity in the relay turbulent channel by using repetition coding.

## 2. System and Channel Model

**Figure 1:**
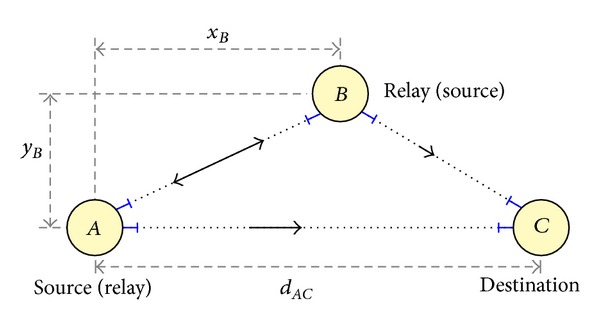
Block diagram of the considered 3-way FSO communication system.

We adopt a three-node cooperative system based on three separate full-duplex FSO links, as shown in Figure 1, assuming intensity-modulated laser sources and ideal noncoherent (direct-detection) receivers.

The cooperative strategy works in three phases or transmission frames, as shown in Table 1. In the first phase, the nodes *A* and *B* send their own data to each other and the destination node *C*; that is, the node *A* (*B*) transmits the same information to the nodes *B* (*A*) and *C*. In the second transmission frame, the nodes *A* and *B* send again the same information to each other delayed by the expected fade duration and, hence, assuming that channel fades are independent and identically distributed (i.i.d.). In the third phase, the node *B* (or *A*) sends the received data from its partner *A* (or *B*) in previous frames to the node *C*.

Following the BDF cooperative protocol, the relay node (*A* or *B*) detects each code bit to “0” or “1” based on repetition coding and sends the bit with the new power to the destination node *C*. When repetition coding is used in the source-relay link transmission during the first and second phases, the information is detected during each transmission frame, combining with the same weight two noisy faded signals. In this way, a diversity gain of two is achieved for this link and, hence, a better performance compared with the traditional BDF cooperative protocol [4, 9]. It must be noted that the symmetry for nodes *A* and *B* assumed in this FSO communication setup and the fact that one transmission frame is overlapped imply that no rate reduction is applied; that is, the same information rate can be considered at the destination node *C* compared to the direct transmission link without using any cooperative strategy. It is here assumed that all the bits detected at the relay are always resent regardless of these bits being detected correctly or incorrectly.

For each link of the three possible links in this three-node cooperative FSO system, the instantaneous current *y*_*m*_(*t*) in the receiving photodetector corresponding to the information signal transmitted from the laser can be written as

(1) $$\begin{array}{c} y_{m}\left(t\right)=\eta L_{m}i_{m}\left(t\right)x\left(t\right)+z_{m}\left(t\right), \end{array}$$

where *η* is the detector responsivity, assumed hereinafter to be the unity, *X*≜*x*(*t*) represents the optical power supplied by the source, *I*_*m*_≜*i*_*m*_(*t*) is the equivalent real-valued fading gain (irradiance) through the optical channel between the laser and the receive aperture, and *L*_*m*_ denotes the deterministic propagation loss. The atmospheric loss *L*_*m*_ is determined by the exponential Beers-Lambert law as *L*_*m*_ = *e* ^−*ψd*^, where *d* is the link distance and *ψ* is the attenuation coefficient. It is given by *ψ* = (3.91/*V*(km))(*λ*(nm)/550)^*q*^, where *V* is the visibility in kilometers, *λ* is the wavelength in nanometers, and *q* is a parameter related to the visibility, with *q* = 1.3 when 6 km < *V* < 50 km. *Z*_*m*_≜*z*_*m*_(*t*) is assumed to include any front-end receiver thermal noise as well as shot noise caused by ambient light much stronger than the desired signal at the detector. It can usually be modeled to high accuracy as AWGN with zero mean and variance *σ* ^2^ = *N*_0_/2, that is, *Z*_*m*_ ~ *N*(0, *N*_0_/2), independent of the on/off state of the received bit. We use *Y*_*m*_, *X*, *I*_*m*_, and *Z*_*m*_ to denote random variables and *y*_*m*_(*t*), *x*(*t*), *i*_*m*_(*t*), and *z*_*m*_(*t*) their corresponding realizations. The irradiance is susceptible to atmospheric turbulence and pointing error effects. It is considered to be a product of two independent random variables; that is, *I*_*m*_ = *I*_*m*_^(*a*)^ *I*_*m*_^(*p*)^, *I*_*m*_^(*a*)^ and *I*_*m*_^(*p*)^ representing the attenuation due to atmospheric turbulence and the attenuation due to geometric spread and pointing errors, respectively. To consider a wide range of turbulence conditions, the gamma-gamma turbulence model proposed in [2] is here assumed. Regarding to the impact of pointing errors, we use the general model of misalignment fading given in [10] by Farid and Hranilovic, wherein the effect of beam width, detector size, and jitter variance are considered. A closed-form expression of the combined probability density function (PDF) of *I*_*m*_ was derived in [11] in terms of the Meijer's *G*-function [12, equation (9.301)]. This PDF appears to be cumbersome to use in order to obtain simple closed-form expressions in the analysis of FSO systems. To overcome this inconvenience, the PDF is approximated by a single polynomial term as *f*_*I*_*m*__(*i*) ≈ *a*_*m*_ *i* ^*b*_*m*_^, based on the fact that the asymptotic behavior of the system performance is dominated by the behavior of the PDF near the origin [9]. Different expressions for *a*_*m*_ and *b*_*m*_, depending on the relation between the jitter variance and turbulence conditions, can be written as

(2a) $$\begin{array}{c} a_{m}=\{\begin{array}{cc} a_{m}^{0}=\frac{\varphi^{2}{\left(\alpha \beta \right)}^{\beta }\Gamma \left(\alpha -\beta \right)}{A_{0}^{\beta }\Gamma \left(\alpha \right)\Gamma \left(\beta \right)\left(\varphi^{2}-\beta \right)}, & \varphi^{2}>\beta ,\\ a_{m}^{1}=\frac{\varphi^{2}{\left(\alpha \beta \right)}^{\varphi^{2}}\Gamma \left(\alpha -\varphi^{2}\right)\Gamma \left(\beta -\varphi^{2}\right)}{A_{0}^{\varphi^{2}}\Gamma \left(\alpha \right)\Gamma \left(\beta \right)}, & \varphi^{2}<\beta , \end{array} \end{array}$$

(2b) $$\begin{array}{c} b_{m}=\{\begin{array}{cc} b_{m}^{0}=\beta -1, & \varphi^{2}>\beta ,\\ b_{m}^{1}=\varphi^{2}-1, & \varphi^{2}<\beta , \end{array} \end{array}$$

where Γ(·) is the well-known Gamma function and *α* and *β* can be directly linked to physical parameters through the following expressions:

(3) $$\begin{array}{c} \alpha ={\left[\exp\left(\frac{0.49\sigma_{R}^{2}}{{\left(1+1.11\sigma_{R}^{12/5}\right)}^{7/6}}\right)-1\right]}^{-1},\\ \beta ={\left[\exp\left(\frac{0.51\sigma_{R}^{2}}{{\left(1+0.69\sigma_{R}^{12/5}\right)}^{5/6}}\right)-1\right]}^{-1} \end{array}$$

with *σ*_*R*_^2^ = 1.23*C*_*n*_^2^ *k* ^7/6^ *d* ^11/6^ being the Rytov variance, which is a measure of optical turbulence strength. Here, *k* = 2*π*/*λ* is the optical wave number, *λ* is the wavelength, and *d* is the link distance in meters. *C*_*n*_^2^ stands for the altitude-dependent index of the refractive structure parameter and varies from 10^−13^ m^−2/3^ for strong turbulence to 10^−17^ m^−2/3^ for weak turbulence [2]. Assuming a Gaussian spatial intensity profile of beam waist radius, *ω*_*z*_, on the receiver plane at distance *z* from the transmitter and a circular receive aperture of radius *r*, *φ* = *ω*_*z*_eq__/2*σ*_*s*_ is the ratio between the equivalent beam radius at the receiver and the pointing error displacement standard deviation (jitter) at the receiver, $\omega_{z_{\text{eq}}}^{2}=\omega_{z}^{2}\sqrt{\pi }\mathrm{erf}(v)/2v\exp(-v^{2})$, $v=\sqrt{\pi }r/\sqrt{2}\omega_{z}$, *A*_0_ = [*erf*⁡(*v*)]^2^, and *erf*⁡(·) is the error function. Hereinafter, the fading coefficient *I*_*m*_ for the paths *A*-*B*, *A*-*C*, and *B*-*C* is indicated by *I*_*AB*_, *I*_*AC*_, and *I*_*BC*_, respectively.

## 3. Error-Rate Performance Analysis

Here, it is assumed that the average optical power transmitted from each node is *P*_opt_, being adopted an on-off keying (OOK) signaling. This scheme is based on a constellation of two equiprobable points in a one-dimensional space with an Euclidean distance of *d*_*E*_ = 2*P*_opt_ *T*_*b*_ *ξ*. Here, the parameter *T*_*b*_ is the bit period and *ξ* represents the square of the increment in Euclidean distance due to the use of a pulse shape of high peak-to-average optical power ratio (PAOPR) [9]. According to (1), the statistical channel model corresponding to the *A*-*B* link assuming repetition coding with EGC during the first and second frames can be written as

(4) $$\begin{array}{c} Y_{AB}=\frac{1}{2}XL_{AB}\left(I_{AB_{1}}+I_{AB_{2}}\right)+Z_{AB_{\text{EGC}}}, \end{array}$$

with *X* ∈ {0, *d*_*E*_} and *Z*_*AB*_EGC__ ~ *N*(0, *N*_0_). Assuming channel side information at the receiver, the conditional BER at the node *B* is given by

(5) $$\begin{array}{c} P_{b}^{AB}\left(E\;|\;I_{AB_{T}}\right)=Q\left(\sqrt{\frac{{\left(d_{E}/2\right)}^{2}i^{2}}{4N_{0}}}\right)=Q\left(\sqrt{\frac{\xi \gamma }{4}}i\right), \end{array}$$

where *I*_*AB*_*T*__ represents the sum of variates *I*_*AB*_*T*__ = *L*_*AB*_(*I*_*AB*_1__ + *I*_*AB*_2__), *Q*(·) is the Gaussian-*Q* function, and *γ* = *P*_opt_^2^ *T*_*b*_/*N*_0_ represents the received electrical SNR in absence of turbulence when the classical rectangular pulse shape is adopted for OOK formats. Since the variates *I*_*AB*_1__ and *I*_*AB*_2__ are independent, knowing that the resulting PDF of their sum *I*_*AB*_*T*__ can be determined by using the moment generating function of their corresponding PDFs, obtained via single-sided Laplace and its inverse transforms, approximate expression for the PDF, *f*_*I*_*AB*_*T*___(*i*), of the combined variates can be easily derived as

(6) $$\begin{array}{c} f_{I_{AB_{T}}}\left(i\right)\approx \frac{a_{AB}^{2}\Gamma {\left(b_{AB}+1\right)}^{2}}{L_{AB}^{2(b_{AB}+1)}\Gamma \left(2b_{AB}+2\right)}i^{2b_{AB}+1}. \end{array}$$

Hence, the average BER, *P*_*b*_^*AB*^(*E*), can be obtained by averaging *P*_*b*_^*AB*^(*E* | *I*_*AB*_*T*__) over the previous PDF [12, equation (6.281)], obtaining the corresponding closed-form asymptotic solution for the BER as can be seen in

(7) $$\begin{array}{c} P_{b}^{AB}\left(E\right)≐\frac{a_{AB}^{2}2^{b_{AB}}\Gamma \left(b_{AB}+1\right)}{L_{AB}^{2(b_{AB}+1)}\left(b_{AB}+1\right)}{\left(\gamma \xi \right)}^{-(b_{AB}+1)}, \end{array}$$

where the value of the parameters *a*_*AB*_ and *b*_*AB*_ depends on the relation between *φ* ^2^ and *β* as obtained in (2a) and (2b), corroborating not only that the diversity order corresponding to the source-relay link is independent of the pointing error when *φ* ^2^ > *β* but also the fact that the diversity order has been increased twice if compared to the traditional BDF cooperative protocol [4, 9]. Once the error probability at the node *B* is known, two cases can be considered to evaluate the BER corresponding to the BDF cooperative protocol here proposed depending on the fact that the bit from the relay *A*-*B*-*C* is detected correctly or incorrectly. In this way, the statistical channel model corresponding to the BDF cooperative protocol, that is, the bits received at *C* directly from *A*-*C* link and from the relay *A*-*B*-*C*, can be written as

(8) $$\begin{array}{c} Y_{\text{BDF}}=\frac{1}{2}XL_{AC}I_{AC}+Z_{AC}+\frac{1}{2}X^{*}L_{BC}I_{BC}+Z_{BC}, \end{array}$$

with *X* ∈ {0, *d*_*E*_} and *Z*_*AC*_, *Z*_*BC*_ ~ *N*(0, *N*_0_/2), where X* represents the random variable corresponding to the information detected at *B* and, hence, X* = *X* when the bit has been detected correctly at *B* and X* = *d*_*E*_ − *X* when the bit has been detected incorrectly. In a similar way to that in [9], but here considering the path loss, we can obtain the corresponding closed-form asymptotic solution for the BER when the bit is correctly detected at *B*, *P*_*b*_^0^(*E*), and when the bit is incorrectly detected at *B*, *P*_*b*_^1^(*E*), as follows:

(9) Pb0(E)≐aACaBC2(bAC+bBC)/2Γ(bAC+1)Γ(bBC+1)LAC(bAC+1)LBC(bBC+1)Γ((1/2)(bAC+bBC+4))×(γξ)−(1/2)(bAC+bBC+2),

(10) $$\begin{array}{c} P_{b}^{1}\left(E\right)≐\int_{0}^{\infty }\int_{0}^{L_{BC}i_{2}/L_{AC}}f_{I_{AC}}\left(i_{1}\right)f_{I_{BC}}\left(i_{2}\right)di_{1}di_{2}. \end{array}$$

Once the error probability at the node *B* is known and considering these two cases, the BER corresponding to the BDF cooperative protocol here proposed is given by

(11) $$\begin{array}{c} P_{b}^{\text{BDF}}\left(E\right)=P_{b}^{0}\left(E\right)\cdot \left(1-P_{b}^{AB}\left(E\right)\right)+P_{b}^{1}\left(E\right)\cdot P_{b}^{AB}\left(E\right). \end{array}$$

This expression can be simplified taking into account the asymptotic behavior previously obtained in (7) and (9) as follows:

(12a) $$\begin{array}{c} P_{b}^{\text{BDF}}\left(E\right)≐P_{b}^{0}\left(E\right),\;\;\;b_{AC}+b_{BC}<2b_{AB}, \end{array}$$

(12b) $$\begin{array}{c} P_{b}^{\text{BDF}}\left(E\right)≐P_{b}^{1}\left(E\right)\cdot P_{b}^{AB}\left(E\right),\;\;b_{AC}+b_{BC}>2b_{AB}. \end{array}$$

Taking into account these expressions, the adoption of the BDF cooperative protocol here analyzed translates into a diversity order gain, *G*_*d*_, relative to the noncooperative link *A*-*C* of

(13) $$\begin{array}{c} G_{d}=\frac{\min\left(2+b_{AC}+b_{BC},2\left(1+b_{AB}\right)\right)}{\left(1+b_{AC}\right)}. \end{array}$$

**Figure 2:**
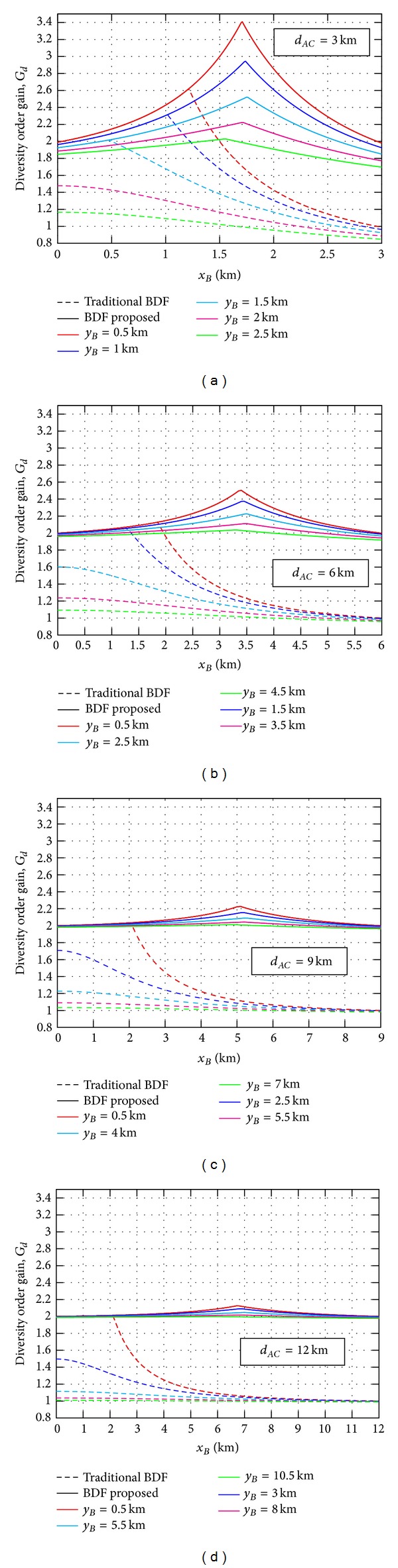
Diversity order gain for source-destination link distances of *d*_*AC*_ = {3,6, 9,12} km when different relay locations are assumed.

Comparing with [9, equation (25)], it must be noted that a factor of 2 is included in relation to the diversity order depending on the source-relay link because of repetition coding. The main aspect to consider in order to optimize the error-rate performance is the relation between *φ* ^2^ and *β* as obtained in (2b) for each link, corroborating that the diversity order is independent of the pointing error when *φ* ^2^ > *β*. Once this condition is satisfied an optimization analysis about (13) is required, evaluating if the diversity order corresponding to the BDF cooperative protocol is determined by the source-destination and relay-destination links or by the source-relay link. For better understanding of the impact of the configuration of the three-node cooperative FSO system under study, the diversity order gain *G*_*d*_ in (13) as a function of the horizontal displacement of the relay node, *x*_*B*_, is depicted in Figure 2 when a wide range of source-destination link distances and different relay locations are assumed. Here, the diversity order gain corresponding to the traditional BDF cooperative protocol [9] is also included in order to show the improvement in performance achieved when repetition coding is assumed in the source-relay link transmission. It is easy to deduce that the traditional BDF protocol only provides cooperative diversity for shorter source-relay distances since the performance is very related to the source-relay link quality, being strongly vulnerable to its turbulence conditions. However, the BDF protocol here proposed presents a greater robustness to the relay node location, allowing for choosing a potential relay node in a greater area while maintaining a high cooperative diversity regardless of the source-destination link distance.

**Figure 3:**
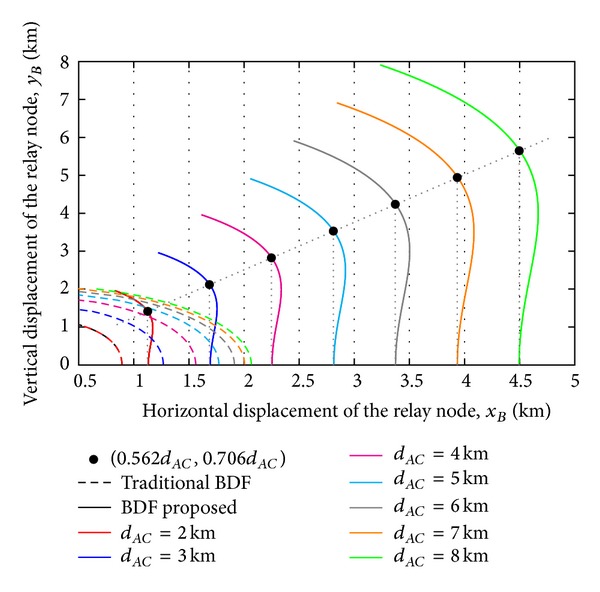
Horizontal relay location wherein the diversity order gain *G*_*d*_ is maximized as the vertical relay location is increased for different source-destination link distances.

Additionally, it can be deduced from (13) that the diversity order gain, *G*_*d*_, is lower as the value of *y*_*B*_ is increased, presenting a maximum value wherein the two profiles related to the expressions (*β*_*AC*_ + *β*_*BC*_)/*β*_*AC*_ and 2*β*_*AB*_/*β*_*AC*_ intersect. A numerical analysis of the location of the maximum value of *G*_*d*_ is displayed in Figure 3 for different relay locations and source-destination link distances from 2 to 8 km.

**Figure 4:**
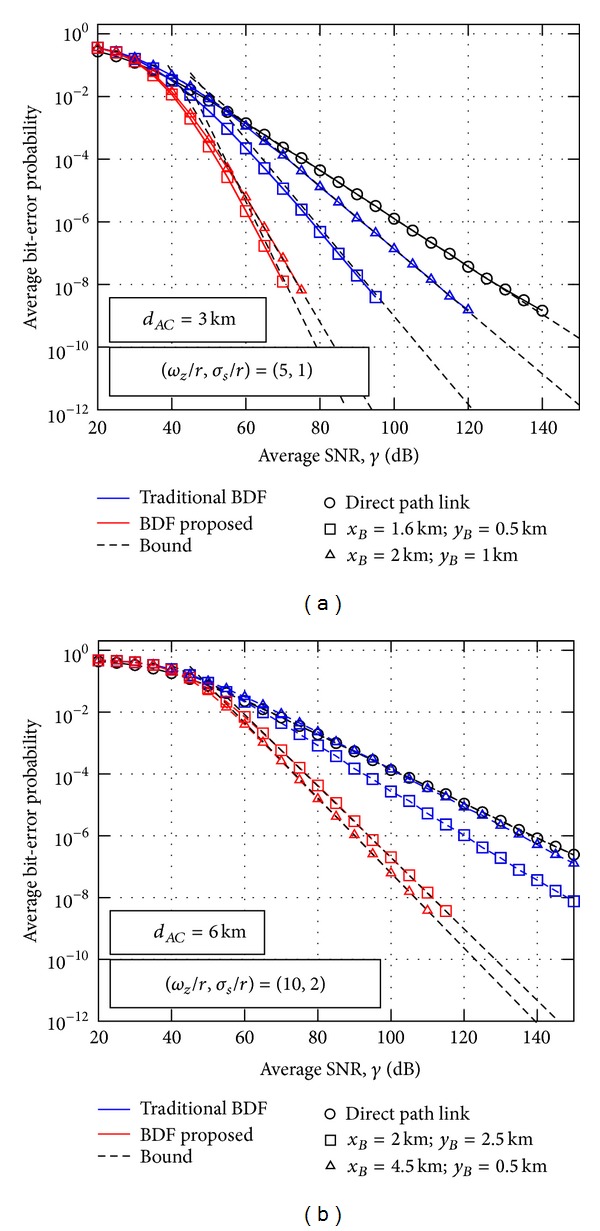
BER performance when different relay locations for *d*_*AC*_ = {3,6} km are assumed together with values of normalized beamwidth and normalized jitter of (*ω*_*z*_/*r*, *σ*_*s*_/*r*) = (5,1) and (*ω*_*z*_/*r*, *σ*_*s*_/*r*) = (10,2), respectively.

In agreement with the results displayed in Figure 2, the location of the maximum value of *G*_*d*_ is proportional to the source-destination link distance, *d*_*AC*_, with an initially increasing value of *x*_*B*_ as the value of *y*_*B*_ is increased. It can be numerically shown that the location of the maximum value of *G*_*d*_ is initially achieved at 56.2% of the source-destination link distance *d*_*AC*_, later increasing this value as *y*_*B*_ is increased until *y*_*B*_ is 70.6% of *d*_*AC*_. Next, as the value of *y*_*b*_ is increased, the maximum value of *G*_*d*_ is located at a value of *x*_*B*_ lower than 0.562*d*_*AC*_. In Figure 3, a similar performance for the BDF cooperative protocol here proposed is corroborated regardless the value of *d*_*AC*_, presenting a more favorable relay location when *x*_*B*_ is close to 0.562*d*_*AC*_ and *y*_*B*_ < 0.706*d*_*AC*_, in excellent agreement with the results displayed in Figure 2 for values of *d*_*AC*_ of 3, 6, 9, and 12 km. Additionally, the performance corresponding to the traditional BDF cooperative protocol is also included in order to corroborate the greater vulnerability to the relay location, showing that its adoption is not advantageous for source-relay distances above 2 km. The improvement in performance can be easily corroborated by the fact that, even when the available diversity order is dependent on the relay location, this is now related to the expression 2*β*_*AB*_/*β*_*AC*_, being twice as in the traditional BDF protocol, fully exploiting the potential time-diversity available in the turbulent channel corresponding to the source-relay link. The results corresponding to this asymptotic analysis with rectangular pulse shapes and *ξ* = 1 are illustrated in Figure 4, when different relay locations for source-destination link distances *d*_*AC*_ = {3 km, 6 km} are assumed together with values of normalized beamwidth and normalized jitter of (*ω*_*z*_/*r*, *σ*_*s*_/*r*) = (5,1) and (*ω*_*z*_/*r*, *σ*_*s*_/*r*) = (10,2). The parameters *α* and *β* are calculated from (3), and values of *λ* = 1550 nm and *C*_*n*_^2^ = 1.7 × 10^−14^ m^−2/3^ are adopted. It must be commented that the impact of the deterministic propagation loss *L*_*m*_ is here considered, assuming clear weather conditions with visibility of 16 km. Monte Carlo simulation results are furthermore included as a reference, confirming the accuracy and usefulness of the derived results. Due to the long simulation time involved, simulation results only up to BER = 10^−9^ are included. Simulation results corroborate that asymptotic expressions here obtained lead to simple bounds on the bit error probability that get tighter over a wider range of SNR as the turbulence strength increases. Additionally, we also consider the performance analysis for the direct path link (noncooperative link *A*-*C*) to establish the baseline performance as well as BER performance corresponding to the traditional BDF cooperative protocol [9]. It can be corroborated that these BER results are in excellent agreement with previous results shown in Figure 2 in relation to the diversity order gain achieved for this 3-way FSO communication setup. In this way, diversity gains of 3.18 and 2.6 are achieved when *d*_*AC*_ = 3 km and relay locations of (*x*_*B*_ = 1.6 km; *y*_*B*_ = 0.5 km) and (*x*_*B*_ = 2 km; *y*_*B*_ = 1 km), respectively, in contrast to the diversity gains of 1.84 and 1.3 are achieved by the traditional BDF cooperative protocol. Analogously, it can be seen that diversity gains of 2.07 and 2.18 are obtained when *d*_*AC*_ = 6 km and relay locations of (*x*_*B*_ = 2 km; *y*_*B*_ = 2.5 km) and (*x*_*B*_ = 4.5 km; *y*_*B*_ = 0.5 km), respectively, in contrast to the diversity gains of 1.31 and 1.09 are achieved by the traditional BDF cooperative protocol. It is corroborated that the traditional BDF protocol only provides cooperative diversity for shorter source-relay distances since its performance is very related to the source-relay link quality, especially when greater source-destination distances are considered. From previous results, it can be concluded that not only a significant improvement in performance has been obtained by increasing the diversity order but also that a greater robustness is now achieved regardless of the source-destination link distance.

## 4. Conclusions

In this paper, a novel bit-detect-and-forward relaying scheme using time-diversity in the relay channel is proposed, significantly improving the robustness to impairments proper to these systems such as unsuitable alignment between transmitter and receiver as well as fluctuations in the irradiance of the transmitted optical beam due to the atmospheric turbulence. Closed-form asymptotic BER expressions are derived for a 3-way FSO communication setup when the irradiance of the transmitted optical beam is susceptible to either a wide range of turbulence conditions or pointing errors. Fully exploiting the potential time-diversity available in the relay turbulent channel, a relevant better performance is achieved, compared with the traditional BDF cooperative protocol [4, 9], showing a greater robustness to the relay location since a high diversity gain is provided regardless of the source-destination link distance. Additionally, asymptotic expressions are used to determine a more favorable relay location.

## Figures and Tables

**Table 1:** BDF cooperative scheme based on repetition coding in the source-relay link transmission.

Symbol	*t*_1_	*t*_2_	*t*_3_	⋯	*t*_*n*_	*t*_*n*+1_	*t*_*n*+2_	*t*_*n*+3_
*A* → *C*								
1	*A* → *B* *A* → *C*			⋯	*A* → *B*	*B* → *C*		
2		*A* → *B* *A* → *C*		⋯		*A* → *B*	*B* → *C*	
3			*A* → *B* *A* → *C*	⋯			*A* → *B*	*B* → *C*
⋮								

*B* → *C*								
1	*B* → *A* *B* → *C*			⋯	*B* → *A*	*A* → *C*		
2		*B* → *A* *B* → *C*		⋯		*B* → *A*	*A* → *C*	
3			*B* → *A* *B* → *C*	⋯			*B* → *A*	*A* → *C*
⋮								
